# Victimization mechanisms and countermeasures in telecom network fraud: a dual-system theoretical perspective

**DOI:** 10.3389/fpsyg.2025.1637935

**Published:** 2025-09-18

**Authors:** Fuming Xu, Anning Liu, Xinjing Li

**Affiliations:** ^1^Faculty of Education, Yunnan Normal University, Kunming, China; ^2^School of Education Science, Nanning Normal University, Nanning, China

**Keywords:** dual-system theory, heuristic bias, telecom network fraud, three-stage dual-process model, decision making

## Abstract

Despite individuals’ awareness of the risks associated with fraud, they frequently engage in irrational decision-making within the realm of telecom network fraud, revealing a dissonance in their cognitive information-processing systems. This paper leverages dual-system theory and the three-stage dual-process model to scrutinize the cognitive trajectories of victims, accentuating the heuristic biases driven by System 1, alongside the ineffective intervention by System 2. Furthermore, it introduces a novel framework that maps “Cognitive Bias, Fraud Type, and Countermeasures.” The study underscores the combined impact of individual characteristics and situational variables in influencing the operation of both cognitive systems, providing insights for the cognitive design of targeted fraud prevention strategies.

## Introduction

Telecom network fraud involves deceptive practices where criminals use text messages, phone calls, and internet tools to manipulate facts or hide the truth. This manipulation leads victims to develop cognitive biases that result in financial losses (Wu, 2021). As artificial intelligence technologies advance, telecom network fraud has become more complex and varied, featuring cross-border operations, anonymity, and organized group structures (Li and Wang, 2023). This evolution makes recovering lost assets particularly difficult. Telecom network fraud has increasingly evolved into a significant global governance challenge. In addition to the direct financial losses incurred, it inflicts psychological harm and engenders broader societal implications, including wasted time, diminished law enforcement resources, and escalating regulatory costs (Bosley et al., 2019). Victims often make suboptimal decisions during critical moments, despite possessing some fraud awareness, ultimately succumbing to sophisticated traps devised by fraudsters. This phenomenon suggests that victimization by fraud is not merely a consequence of information asymmetry; rather, it is deeply entrenched in individual cognitive processing failures. Fraudsters deliberately construct high-pressure, ambiguous, and urgent scenarios to elicit fast, low-reflection thinking, thereby undermining traditional risk assessment mechanisms. A nuanced understanding of how individuals process information in contexts of fraud and the reasons for deviations from rational judgment is essential for the development of effective and targeted anti-fraud strategies. To enhance the practical relevance of the theoretical framework presented, the subsequent sections will illustrate each cognitive bias with concrete examples drawn from real-world telecom fraud scenarios.

Existing research on telecom network fraud victimization primarily operates at three analytical levels. The first level examines demographic characteristics, such as age, income, gender, and occupational type (Luo and Zhang, 2021). The second level focuses on psychological traits, including personality dimensions, emotional arousal, and trust propensity (Tang and Wang, 2016; Xie, 2022). The third level investigates cognitive mechanisms, particularly emphasizing heuristic biases (Jones et al., 2019; Vishwanath et al., 2018). While these findings have advanced the theoretical understanding of victimization, they remain fragmented in their explanation of the underlying mechanisms. Most studies adopt a static perspective, merely describing the characteristics of victims’ cognitive errors, yet they overlook the dynamic information-processing mechanisms and the interactive functioning of cognitive systems during decision-making related to fraud.

The dual-system theory offers a critical framework for analyzing irrational decision-making. This theory posits that human judgment is governed by two cognitive systems: System 1, which is characterized as fast, intuitive, and efficient, yet susceptible to heuristic biases arising from contextual cues; and System 2, which operates more slowly, engages in analytical thought, and demands greater cognitive resources. System 2 can inhibit intuitive responses and facilitate systematic reasoning (Kahneman and Frederick, 2002; Evans, 2008). In the context of fraud, individuals predominantly rely on System 1 for their initial judgments. When System 2 fails to engage effectively, the likelihood of irrational decision-making increases. However, existing research applying dual-process theory often adopts a static dichotomy, framing the issue as “System 1 error versus System 2 failure to correct,” and lacks a nuanced account of how the two systems dynamically interact, compete for cognitive resources, and detect conflict within specific fraudulent contexts.

To address this limitation, the present study introduces the three-stage dual-process model of analytic engagement (Pennycook et al., 2015a), which conceptualizes fraud-related decision-making as comprising three sequential stages: an initial intuitive response driven by System 1, a conflict detection stage, and the subsequent engagement of System 2. This model emphasizes the heuristic biases induced by System 1, and underscores the importance of conflict detection, as well as the extent to which System 2 can effectively inhibit or override erroneous intuitions. This dynamic cognitive approach elucidates why individuals may still succumb to fraud, even when they possess an awareness of potential risks. Additionally, this study proposes a “Cognitive Bias–Fraud Type–Countermeasures” mapping framework, designed to bridge theoretical mechanisms with practical intervention strategies. Rather than introducing new theory or empirical data, this review aims to offer an applied synthesis of existing dual-process models to support research, intervention, and policy design in the context of telecom network fraud.

## Cognitive mechanisms of fraudulent decision-making

### Heuristic processing and cognitive biases under the dominance of system 1

The cognitive processing of System 1 is characterized by automaticity and unconscious operation, enabling the rapid and efficient handling of information. It primarily relies on intuition and prior experience, allowing individuals to swiftly detect potential threats. However, due to its dependence on limited cognitive resources, System 1 is also susceptible to systematic errors, which can frequently result in biased decision-making.

#### Representativeness heuristic

The representativeness heuristic is a cognitive strategy in which individuals judge probabilities based on the similarity between specific features and a prototypical category. This heuristic often leads individuals to neglect the base rates of events (Kahneman, 2011), resulting in biased evaluations. In telecom network fraud, deceptive information typically constructs seemingly credible sources—such as familial relationships or authoritative institutions—and incorporates specific personal details of the victim, thereby impairing rational judgment. Empirical research by Luo et al. (2013) indicates that recipients’ susceptibility to fraudulent information is associated with the perceived credibility of the information source and the density of embedded details. Studies on online auction fraud demonstrate that website characteristics significantly influence individuals’ trust judgments regarding transactions, serving as a key factor in economic losses from e-commerce fraud (Zhang et al., 2018). A typical example of fraudsters exploiting the representativeness heuristic is found in scams impersonating public security authorities. In such cases, the use of specialized terms like “security account review” aligns with the public’s cognitive schema of authoritative institutions. This typicality matching with authority symbols induces a psychological set of institutional compliance, prompting individuals to overlook the base probability of anomalous requests.

#### Availability heuristic

The availability heuristic is a cognitive mechanism by which individuals estimate the likelihood of an event based how easily related information can be retrieved from memory and the vividness of mental simulation (Kahneman, 2011). Carroll (1978) experimentally demonstrated that participants rated the probability of imaginable events significantly higher than those control groups, confirming a positive correlation between psychological availability and subjective probability judgments. In phishing contexts, individuals who already expect certain types of information are more likely to respond to phishing emails (Jayatilaka et al., 2024). The availability heuristic shapes risk judgment by the number of instances that come readily to mind (Efendić, 2021). For example, in pyramid scheme fraud, participants often rationalize their involvement and avoid discussing losses; consequently, others exposed to them primarily recall frequently retrieved cues, such as success stories or earnings evidence actively promoted by the scheme. This reliance on accessible, positively framed examples increases susceptibility to fraud (Bosley et al., 2019). Moreover, the availability heuristic directs individuals’ attention toward short-term high-yield gains (Xie et al., 2023), partially explaining why individuals fall for profit traps in telecom network fraud. Fraudulent messages are often crafted around socially salient topics and recent events, making the scam content more imaginable and, therefore, more believable. For instance, at the beginning of the school term, fraudsters may send messages such as: “Notice from the Ministry of Education: Your child qualifies for a scholarship. Click the link to fill out the information and receive educational funding,” Such messages exploit vivid and timely associations to trigger availability-based judgments.

#### Anchoring and adjustment

Anchoring and adjustment, also known as the anchoring effect, refers to a decision-making heuristic in which individuals rely on an initial anchor—derived from the framing of a problem or prior experience—as a reference point for subsequent numerical adjustments. Due to systematic under-adjustment, this process frequently results in biased judgments (Kahneman, 2011). The anchoring effect is pervasive across diverse decision-making contexts. Individuals exposed to high anchor values typically exhibit greater difficulty adjusting their judgments downward (Furnham and Boo, 2011). Under conditions of time pressure, the adjustment process is further compressed, resulting in minimal downward corrections and an intensification of judgment bias (Yik et al., 2019). In context of financial fraud, for example, perpetrators frequently establish a high anchor by asserting “expected annualized returns of 30–50%” thereby constructing a deceptive investment framework. By manipulating the presentation of returns, these fraudsters encourage ongoing investments from victims, ultimately culminating in the fraud scheme when they freeze the victims’ funds (Ye, 2023). In this scenario, the promised return 30–50% acts as a strong reference anchor, significantly influencing victims’ evaluations of potential returns. As a result, even when faced with negative indicators, such as delays in withdrawal, victims may disregard these warning signs due to the compelling nature of initial high-return promise.

#### Affect heuristic

The affect heuristic is a cognitive phenomenon in which individuals attribute varying degrees of emotional valence to their mental representations of objects and events, thus influencing their decision-making processes (Slovic et al., 2007). A key aspect of affective decision-making is the inverse relationship between perceived risk and perceived benefit. Specifically, an increase in the subjective evaluation of benefits is associated with a decrease in risk perception (Watson et al., 2017). In positive emotional states, individuals show heightened sensitivity to anticipated gains while allocating fewer attentional resources to potential risks (Slovic and Peters, 2006). Fraudsters frequently exploit this cognitive bias by embedding positive emotional cues, such as significant material rewards, to manipulate victims’ assessments of risk and benefit. In cases of “romance scam,” for example, perpetrators often express concern and offer compliments during conversations, providing victims with substantial “emotional value” (Xiang and Liu, 2021). Furthermore, research demonstrates that both older and younger individuals are more vulnerable to misleading advertisements when experiencing heightened emotional arousal, leading to poor purchasing decisions (Kircanski et al., 2018). This susceptibility arises because individuals prone to affect heuristic processing experience greater emotional fluctuations during risk-related tasks, resulting in cognitive overload and prompting rapid, less deliberative decisions (Miao and Chi, 2022).

### Conflict monitoring

According to the conflict monitoring theory articulated by Botvinick et al. (2004), individuals are able to detect discrepancies between the intuitive responses produced System 1 and the analytical reasoning of System 2. When such a conflict arises, individuals can activate additional cognitive control resources to address it. However, there are contexts in which the conflict detection mechanism may fail to engage effectively. Research conducted by De Neys et al. (2008) suggests that while individuals may experience a vague awareness of conflict when presented with contradictory information, this detection mechanism may not operate optimally due to limitations in the cognitive resources of System 2 or insufficient allocation of allocation. Within the context of telecom network fraud, victims may be unable to identify or respond to conflict cues due to factors such as time pressure, heightened emotional arousal, or overabundance of trust in the information source. For instance, under urgent conditions, individuals may neglect logical inconsistencies or aspects of fraudulent communications, resulting in a breakdown of the conflict detection process.

### Engagement pathways of system 2: rationalization and cognitive decoupling

If a conflict is detected during the second stage of decision-making, System 2 is activated in the third stage to facilitate analytical processing. At this point, two types of cognitive processing may occur: rationalization and cognitive decoupling (Pennycook et al., 2015a). Rationalization involves a process where individuals, despite recognizing the conflict, attempt to justify their initial intuitive response. For example, a victim of a scam may continue to invest, reasoning, “Just one more payment, and I’ll recover my principal.” In such cases, individuals may be aware of the inconsistency between their actions and their original goals yet still find it difficult to disengage from the fraudulent scheme. In contrast, cognitive decoupling entails the suppression and replacement of intuitive outputs generated by System 1. When individuals engage in cognitive decoupling, they can temporarily inhibit their intuitive judgments, allowing for a more thorough analysis and evaluation, thereby reducing the risk of deception. The successful execution of cognitive decoupling relies on essential functions of System 2, including cognitive reflection, analytical reasoning, and executive control, which enable individuals to override their initial intuitions and engage in more deliberate and effortful information processing.

#### Cognitive reflection

Cognitive reflection is defined as the ability to override intuitive responses in order to arrive at normatively accurate conclusions (Primi et al., 2016). Originally conceptualized by Frederick (2005) and subsequently operationalized through the Cognitive Reflection Test (CRT), this construct serves as a fundamental aspect of System 2 thinking and has emerged as a significant predictor in the investigation of decision-making mechanisms among victims of telecom network fraud (Mosleh et al., 2021). Research conducted by Ackerley et al. (2022) revealed that individuals with elevated levels cognitive reflection demonstrate enhanced cue integration efficiency and improved decision accuracy in tasks designed to detect phishing attempts. Conversely, individuals with lower cognitive reflection proficiency exhibit suboptimal performance in these tasks, likely due to an overreliance on intuitive processing—a cognitive style that increases the likelihood of failing to adequately filter critical information (Jones et al., 2019). Moreover, cognitive reflection is intricately linked to online behavior, particularly within social media platforms that serve as significant conduits for digital fraud (Vishwanath, 2015a,b). Findings by Mosleh et al. (2021) indicate that individuals possessing high cognitive reflection are more inclined to verify the authenticity of information, whereas those with lower cognitive reflection are characterized by increased gullibility and heightened susceptibility to scams. Furthermore, case studies examining internet-based fundraising fraud suggest that individuals with greater cognitive reflection engage in more systematic risk–benefit analyses and employ more comprehensive information processing during their decision-making processes (Wu et al., 2022).

#### Analytical reasoning

Analytical reasoning encompasses the cognitive process through which individuals systematically assess a range of potential options and outcomes in the context of decision-making. This mode of reasoning diverges from System 1 processes, which are characterized by reliance on superficial information extraction, as analytical reasoning entails necessitates a deliberate and effortful engagement with available data to formulate coherent and well-supported conclusions (Stanovich, 2015). This cognitive ability is paramount in the identification of pseudo-profound nonsense—statements that, despite appearing meaningful at a glance are ultimately vague, logically inconsistent, and devoid of substantive content (Pennycook et al., 2015b). The functionality of analytical reasoning can be compared to the capability to discern sophisticated phishing websites: while these fraudulent sites may closely replicate the aesthetic elements of legitimate online platforms, they harbor significant risks associated with information theft. A comparative study conducted by Kelley et al. (2023) illustrated that individuals exhibiting stronger analytical reasoning skills demonstrated a significantly higher accuracy in detecting spoofed websites in contrast to those who primarily relied on intuitive judgments, thereby reducing their susceptibility to online fraud.

#### Executive function

Executive function is defined as an individual’s ability to regulate and oversee numerous cognitive processes during intricate tasks, ultimately aimed at fostering goal-directed and coordinated behavior (Zhou, 2004). The three-component model established by Pennington and Ozonoff (1996) identifies three fundamental dimensions of executive function: working memory, inhibitory control, and cognitive flexibility. Working memory, which is responsible for the temporary retention and manipulation of information, serves as the cornerstone of executive function. Inhibitory control refers to the capacity to actively suppress distracting stimuli while remaining focused on task objectives, whereas cognitive flexibility entails the psychological ability to modify cognitive strategies in accordance with shifting situational demands (Li et al., 2006). In the context of telecom network fraud, such fraudulent activities can be viewed as sophisticated decision traps orchestrated by perpetrators, particularly through targeted scams that exploit sensitive personal information. This scenario imposes significant demands on individuals’ executive functioning, as effective avoidance of victimization necessitates rational decision-making through cognitive regulation supported by robust executive functions. Müller et al. (2021) highlighted a correlation between executive function and individuals’ propensity to make advantageous decisions in risk-related tasks, noting that those with diminished executive functioning encounter heightened challenges and increased error rates in risky decision-making. Furthermore, the caliber of executive function plays a pivotal role in individuals’ ability to detect deception. Gavett et al. (2017) found that individuals exhibiting higher executive function are less susceptible to phishing attacks. This relationship may be moderated by the connection between executive function and probabilistic reasoning skills, suggesting that enhanced numerical processing abilities in risk decision-making contribute to improved evaluative accuracy (Brand et al., 2014).

**Figure 1:**
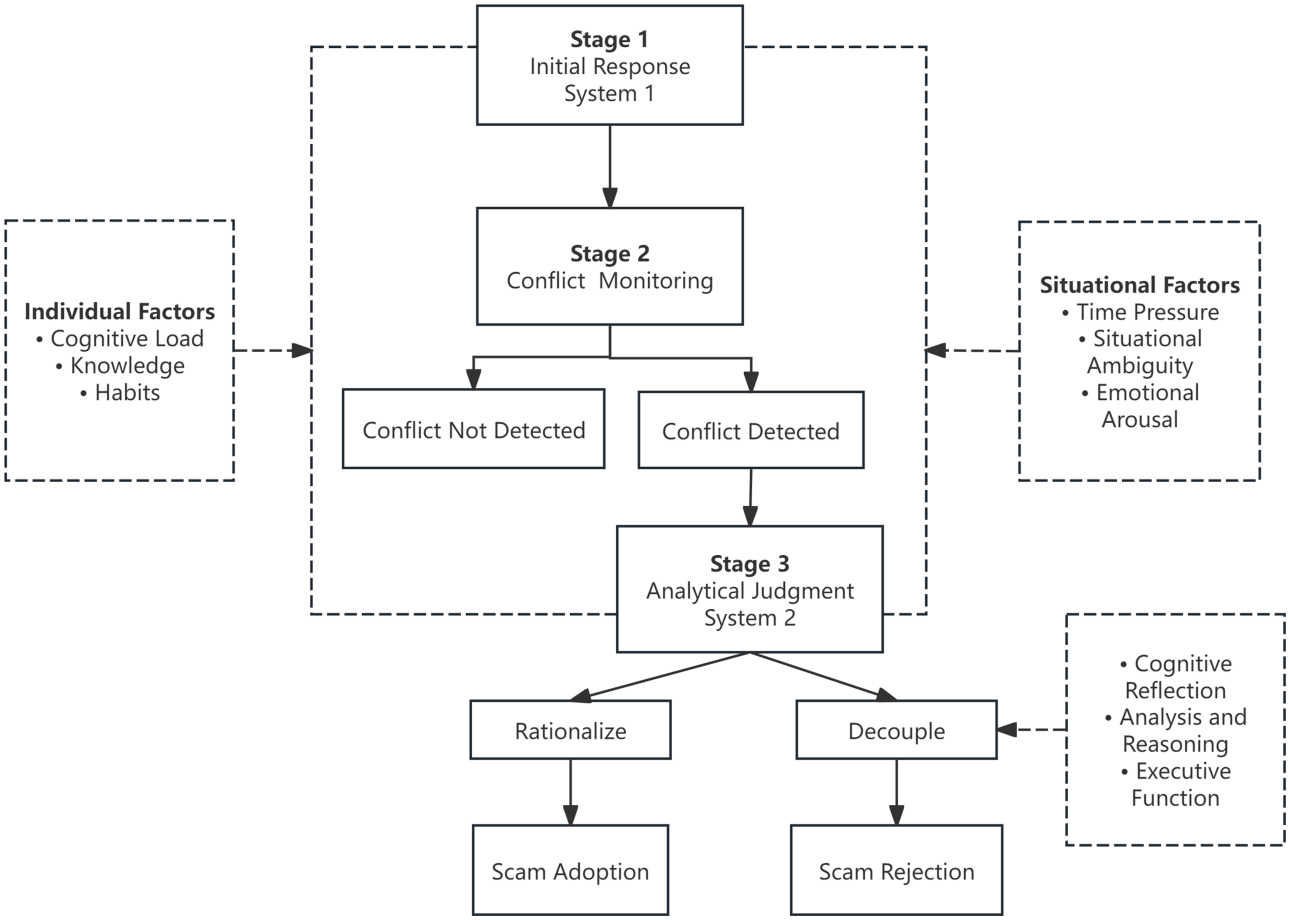
Cognitive process of victims of telecom internet fraud.

In conclusion, cognitive biases in fraud-related decision-making emerge not from the dysfunction of an isolated cognitive system, but rather from a dynamic imbalance between System 1 and System 2. Utilizing the three-stage dual-process model of analytic engagement (Pennycook et al., 2015a), the cognitive processing pathways of victims within telecom network fraud (Figure 1) can be articulated as follows: During the initial stage, System 1 rapidly governs judgment through heuristics, which facilitate the onset of initial cognitive biases. In the second stage, should the conflict detection mechanism fail to activate adequately, any discrepancies between intuitive responses and objective reality may be disregarded. This oversight can hinder timely intervention by System 2. In the final stage, even if System 2 is engaged, effective correction of errors may be obstructed by factors such as limited cognitive resources, motivated reasoning, or diminished executive function. Consequently, this can lead to the reinforcement of erroneous judgments through rationalization, rather than facilitating accurate appraisal. Conversely, if individuals achieve an awareness of their intuitive biases and manage to rectify them through cognitive decoupling, the fraudulent process may be interrupted, thereby mitigating the risk of victimization.

## Factors influencing dual-system information processing and countermeasures

While the cognitive attributes of System 1 and System 2 have been discussed in previous literature, it is crucial to delve deeper into how their interaction is influenced by various individual and situational factors. At the individual level, elements such as cognitive load, knowledge, and media usage habits play significant roles. Additionally, contextual pressures—including time constraints and environmental ambiguity—further impact the delicate equilibrium between intuitive and deliberative reasoning, particularly in the context of fraud scenarios. Understanding these dynamics is essential for a comprehensive analysis of decision-making processes in such situations.

### Individual factors

Within the dual-process framework, individual characteristics function as significant moderators in the allocation of cognitive resources and the activation of System 1 and System 2 processing. First, cognitive load serves as a foundational prerequisite for engaging System 2. Research by Zucchelli et al. (2025) has experimentally demonstrated that participants subjected to cognitive load experience greater depletion of cognitive resources, which impairs their ability to engage System 2 for detailed information processing during risk decision-making tasks. This impairment is evidenced by shorter decision times and an increased reliance on heuristic-driven shortcut strategies, subsequently accelerating risk-taking behavior (He and Jin, 2010). Second, the extent of an individual’s knowledge reserves is critical in determining System 2’s capacity for anomaly detection and intervention. Individuals with extensive domain knowledge are more likely to activate System 2 when recognizing informational anomalies and are better positioned to correct initial System 1 judgments through logical reasoning, thereby enhancing decision accuracy. Conversely, a deficit in knowledge exacerbates dependence on heuristic processing, leading to increased susceptibility to fraudulent activities (Kitchen et al., 2014). Furthermore, long-established habits of digital media usage shape information processing tendencies. For instance, when individuals receive information through familiar platforms such as WeChat or TikTok, they tend to adopt a “default trust” mindset, resulting in an automatic acceptance of content while diminishing their vigilance and motivation to verify the information (Vishwanath, 2015a,b; Vishwanath et al., 2018). Collectively, these three categories of individual-level factors dynamically regulate the interplay between System 1 and System 2, ultimately influencing the likelihood of successful System 2 in decision-making processes.

### Situational factors

In addition to individual factors, specific situational variables substantially influence the processing modes and resource allocation between the dual systems, thereby modulating the extent of cognitive biases within fraud contexts. Among these situational variables, time pressure serves as a particularly salient form of external interference. Under urgent decision-making conditions, individuals often encounter difficulties in engaging in the in-depth analytical processes of System 2 and instead predominantly rely on System 1’s rapid responses. This reliance can lead to compressed information filtering and judgment processes, which markedly increase error rates (Kahneman, 2011; Suri and Monroe, 2003). Furthermore, situational ambiguity exacerbates informational uncertainty, impairing risk identification and hindering individuals’ ability to formulate clear judgments regarding the content at hand. This condition typically results in a heightened dependence on heuristic strategies (Huang et al., 2014). In fraud scenarios, ambiguity frequently emerges through the use of vague language and a confluence of truthful and deceptive information, strategies that are deliberately employed to undermine System 2’s motivation to intervene effectively. Emotional arousal represents another critical factor that warrants attention. Empirical research has shown that states of high arousal not only restrict individuals’ focus on pertinent informational details but also increase impulsivity in decision-making, thereby elevating the likelihood of irrational judgments (Ye et al., 2023). Within scam interactions, such emotional states are frequently strategically manipulated to suppress reflective thinking and elicit rapid compliance behaviors, exemplified by fabricated winning notifications. Collectively, these situational variables act as external triggers for decision errors in fraud contexts by impacting the availability of cognitive resources and levels of alertness.

### Countermeasures

#### Heuristic biases and countermeasures

In the context of telecom network fraud, perpetrators strategically exploit heuristic biases to manipulate judgments of victims, leading to decisions that lack rational foundation. First, the representativeness heuristic prompts individuals to evaluate the credibility of information based on its perceived alignment with established prototypes or stereotypes. Fraudsters often enhance credibility by impersonating authoritative institutions, such as banks or public security agencies. To counteract this cognitive bias, it is imperative to establish mechanisms for multi-source information verification, thereby reducing reliance on singular sources of information. Moreover, the promotion of foundational education regarding base rates can substantially mitigate individuals’ susceptibility through early warning interventions (Scheibe et al., 2014). The availability heuristic presents another significant cognitive bias, wherein individuals form judgments predominantly based on information that is readily retrievable from memory. Scammers frequently exploit this tendency by repeatedly presenting victims with specific scenarios to shape their decision-making processes. Interventions aimed at disrupting the availability of such information are essential, for instance, the implementation of mandatory cooling-off periods and the reinforcement of official channels for information dissemination can aid individuals in avoiding decisions influenced solely by easily accessible cues. Additionally, anchoring and adjustment biases contribute to the phenomenon, causing victims to place undue weight on initial information—often characterized by attractive promises of high returns. Scammers harness this bias by establishing persuasive initial anchors. To mitigate the effects of anchoring, enhancing risk recognition capabilities is crucial. This may entail the introduction of third-party risk assessment tools, clarifying the latent risks embedded within anchoring scripts, and reinforcing public educational initiatives to foster a sense of vigilance and skepticism when confronted with preliminary information. Lastly, the affect heuristic compels victims to make hasty decisions that are influenced by emotional states— such as fear, anger, or sympathy. To mitigate the adverse impact of emotions on decision-making processes, the implementation of training in emotional regulation and psychological defense strategies is critical. Such training equips individuals to maintain composure during heightened emotional states, thereby minimizing the likelihood of emotion-driven errors. To further elucidate the logical connections among cognitive biases, types of fraud, and corresponding intervention measures, a mapping framework is constructed based on the above analysis (Table 1), providing a reference for designing effective preventive strategies.

**Table 1:** Mapping framework of cognitive biases – types of fraud – countermeasures.

Cognitive bias	Typical fraud type	Countermeasures
Representativeness heuristic	Impersonating public security authorities	Multi-source information verification Base-rate education
Availability heuristic	Scams exploiting hot topics	Mandatory cooling-off periods Alerts for emerging fraud patterns
Anchoring and adjustment	Financial investment fraud	Third-party risk assessment Exposure of anchoring scripts
Affect heuristic	Romance fraud	Emotional regulation techniques Psychological defense strategies

#### Training interventions to mitigate system 2 deficits

In telecom network fraud decision-making, the non-utilization or insufficient effectiveness of System 2 often stems from a combination of excessive cognitive load, inadequate executive function, and the influence of motivated reasoning. High cognitive load conditions hinder individuals from engaging in the analytical processes characteristic of System 2, resulting in an over-reliance on System 1’s intuitive judgments, thereby increasing the risk of victimization. To counteract these tendencies, it is imperative to reduce cognitive load and implement effective decision-support tools. Strategies such as simplification of information presentation and the minimization of multitasking distractions can significantly enhance the engagement of System 2. Moreover, deficits in executive function pose significant challenges to individuals’ regulatory control, particularly in the context of complex decision-making scenarios, where manipulative information is present. Under such conditions, individuals often find it difficult to inhibit intuitive responses that may lead to suboptimal outcomes. Cognitive training interventions designed to improve attention and working memory can bolster the operational capacity of System 2, facilitating more effective risk analysis. Lastly, motivated reasoning often causes individuals to selectively accept judgments congruent with their desires while disregarding potential risks. Reflective thinking training can assist individuals in identifying their cognitive biases and mitigating the adverse effects of motivated reasoning, thereby enhancing the overall quality of rational decision-making.

## Future directions

Grounded in dual-process theory and informed by the three-stage dual-process model, this review highlights three critical factors contributing to irrational decisions: the dominance of heuristics, failures in conflict detection, and insufficient engagement of System 2. However, the empirical evidence in this area remains limited, and the efficacy of intervention strategies has yet to be thoroughly explored. Future research may benefit from the following directions:

### Advancing process modeling of dual-system interaction

Most existing studies adopt a binary distinction between System 1 and System 2, overlooking the dynamic interplay between the two systems in the context of fraudulent decision-making. Future research could expand upon the three-stage model proposed by Pennycook et al. (2015a) by incorporating variables such as cognitive resource allocation, temporal processing windows, and levels of arousal. This approach would facilitate the development of dynamic interaction models that simulate how the two systems are triggered and compete across different fraud scenarios, thus addressing paradoxical cases where individuals recognize risk but still make erroneous decisions.

### Developing ecologically valid experimental paradigms

Current empirical studies often rely on vignette-based or questionnaire methods, lacking the capacity to capture the real-time dynamics of fraud decision-making. Future research should consider leveraging immersive technologies such as virtual reality (VR) and covert online chat tasks, to construct more authentic fraud scenarios such as romantic scams. These could be enhanced by physiological measurements —such as skin conductance and heart rate variability—as well as process-tracing indicators including reaction time, and eye-tracking data. This multidimensional approach would facilitate the identification of behavioral markers indicative of System 1 dominance and System 2 engagement, thereby enhancing ecological validity and generalizability.

### Promoting interaction-based interventions targeting individual and situational factors

Current intervention strategies predominantly focus on addressing individual biases in isolation, often neglecting a systematic analysis of the interplay between personal traits and situational factors. Future research should adopt a person–situation fit perspective, with the objective of developing adaptive intervention tools that are tailored to both types of risk and cognitive profiles. For instance, the implementation of intelligent risk alert systems informed by cognitive styles, as well as decision aids designed to mitigate the effects of emotional priming, could be explored and validated through longitudinal studies assessing their long-term efficacy and transferability.

## Conclusion

This review synthesized existing research on the cognitive mechanisms underpinning telecom network fraud through the lens of dual-process theory and the three-stage dual-process model. It delineates a conceptual pathway wherein heuristic dominance, failure in conflict detection, and inadequate engagement of System 2 processes collectively contribute to the irrational decision-making exhibited by victims. By mapping key cognitive biases to decision-making errors and identifying individual and situational moderators, the review provides a structured framework for understanding susceptibility to fraud.

The proposed framework holds practical implications for the prevention and intervention of fraud. It can inform the development of cognitive training programs designed to enhance conflict detection and executive control functions, particularly for individuals characterized by low cognitive reflection or high exposure to digital environments. Additionally, it may guide the development of fraud detection systems that integrate psychological cues—such as decision-making speed and emotional content—as indicators of heuristic vulnerability. Furthermore, it offers a foundation for public policy innovations, including adaptive warnings, interface nudges, and targeted education campaigns tailored to align with specific individual and situational contexts.

While this review does not propose a novel theoretical model or present new empirical findings, it seeks to integrate and apply established cognitive frameworks within the domain of telecom network fraud. Its contribution, therefore, resides in achieving pedagogical clarity and practical synthesis rather than theoretical innovation. Future research is encouraged to empirically test and extend the proposed framework, leveraging real-world behavioral data, physiological markers, and longitudinal assessments of intervention outcomes.

## Data Availability

No datasets were generated or analysed during the current study. All data discussed or cited are available from the corresponding publications referenced in the article.
